# Er^3+^-Ions-Doped Multiscale Nanoprobes for Fluorescence Imaging in Cellular and Living Mice

**DOI:** 10.3390/nano11102676

**Published:** 2021-10-12

**Authors:** Cong Cao, Yu Xie, Shi-Wen Li, Chang Hong

**Affiliations:** Institute of Smart Biomedical Materials, School of Materials Science and Engineering, Zhejiang Sci-Tech University, Hangzhou 310018, China; kylino370@sina.com (Y.X.); lswww2021@163.com (S.-W.L.); 18892681558@163.com (C.H.)

**Keywords:** upconversion, near-infrared, fluorescence probe, surface modification

## Abstract

With the development of biotechnology, luminescent nanoprobes for biological disease detection are widely used. However, the further application in clinic is limited by the reduced penetration depth in the tissues and light scattering. In this work, we have synthesized NaYF_4_:Yb,Er,Ce@SiO_2_-OAlg nanomaterials, which have both upconversion and near-infrared (NIR) luminescence. The optimized probes were determined to achieve cell imaging by its upconversion (UCL) luminescence and in vivo imaging through collection of NIR fluorescence signals simultaneously. The research is conducive to developing accurate diagnostic techniques based on UCL and NIR fluorescence imaging by a single nanoparticle.

## 1. Introduction

Optical imaging is currently very attractive for in vivo imaging because of its high spatial and temporal resolution [1,2,3]. Limited by emission wavelength and response ability, the development of specific quantifiable fluorescent probes is still a critical challenge [4]. Although significant luminescence intensity can be observed at the cellular level, its development is limited because the penetration depth will be reduced in vivo [5,6,7]. To overcome the difficulties, it is necessary to adjust the position of the emission and absorption bands of the material, and try to bring them within the transparent biological window [8]. The second near-infrared (NIR-II) luminescence for bioimaging reveals higher resolution in deeper tissue [9,10,11,12,13]. However, few probes are able to achieve dual fluorescence imaging at both the cell level and the living organism level [14,15]. Combined with the advantages of multi-peak emission and excellent stability, lanthanide-doped luminescent nanomaterials provide new solutions. Er^3+^-based nanoparticles have been reported as prospective probes, owing to their upconversion emissions at 540 nm and 660 nm and near-infrared emission at 1530 nm.

Actually, the surface modification of the nanoprobes plays an important role to determine the application. After surface modification, the hydrophilic nanoprobes can be achieved from usual oleic capped samples [16]. Silanization is a popular technique for surface modification of lanthanide-doped nanoparticles, since silica is highly biocompatible and easily processed [17,18,19,20]. One of the most salient advantages is the multi-ligands for the conversion of SiO_2_ (e.g., -COOH, -NH_2_, -SH, etc.), which allows for bioapplications [21,22,23]. Furthermore, the silica-coated functional nanoparticles can be protected by the SiO_2_ shell against the influence of physiological conditions and surroundings [24]. Another route is the reverse microemulsion (water-in-oil) method to coat silica with hydrophobic capping ligands. This strategy can obtain silica layers with varying thicknesses. It is easier to co-ordinate with other surface functional groups [25]. The obtained multifunctional nanocomposites can be further used as drug delivery carriers and applied for imaging [26,27,28].

**Figure 1:**
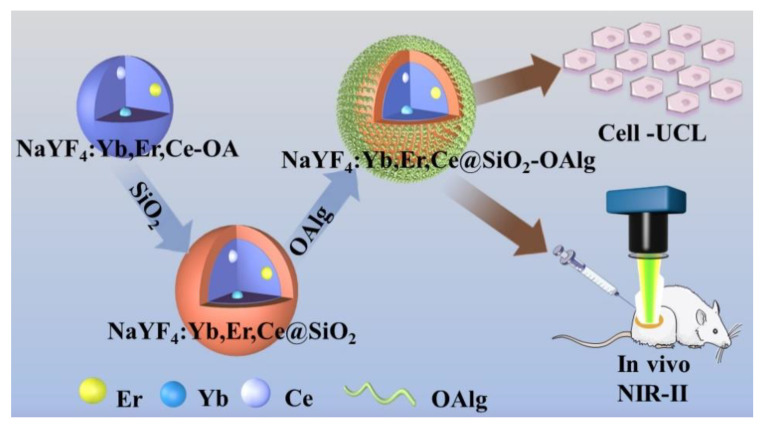
Scheme of the surface modification process of lanthanide luminescent nanoparticles by assisting with SiO_2_ and OAlg molecules.

This project focuses on the construction of multi-scale fluorescent nanoprobes to broaden biological applications (Figure 1). Herein, Er^3+^-based rare earth-doped nanomaterials in the hexagonal phase were synthesized by the hydrothermal method. Then, they were coated with silicon and coupled with oxidized sodium alginate (OAlg) molecules (Figure S1). After coating a layer of silica, the hydrophilicity and upconversion efficiency of nanomaterials can be greatly improved. Here, we coated a layer of sodium alginate to improve biocompatibility [29]. The upconversion and near-infrared luminescence intensity of such nanoparticles were regulated by doping with Ce^3+^ in different proportions. The effects of imaging in cellular UCL and living level NIR-II were collected and compared. Finally, the obtained probes were injected into mice through the caudal vein for luminescent images.

## 2. Materials and Methods

### 2.1. Materials and Characterization

Rare earth chloride and all the solvents were bought from Shanghai Yongsheng Chemical Co. Ltd. (Shanghai, China), including YCl_3_ (>99.99%), YbCl_3_ (>99.99%), ErCl_3_ (>99.99%), CeCl_3_ (99.99%), oleic acid (OA, >90%), 1-octadecene (ODE, >90%), CH_3_OH, EtOH, cyclohexane, CH_2_Cl_2_, and DMSO. NaOH, NH_4_F, sodium periodate (NaIO_4_), sodium alginate, and fetal bovine serum (FBS) solutions were purchased from Adamasbeta Co., Shanghai, China. All the materials were used without further purification.

All TEM images of the nanoparticles were carried out by a JEM-2100 transmission electron microscope (JEOL, Tokyo, Japan). The upconversion emission spectra were determined on a Horiba FluoroMax-4 Spectrometer (Horiba, Kyoto, Japan). The NIR fluorescence spectra were determined on a FLS920 Luminescence Spectrometer (Edinburgh, England). The FTIR data were recorded by a Niclot-5700 Fourier Transform Infrared Spectrometer (Thermo Fisher, Waltham, MA, USA). The XRD patterns of the nanoparticles were determined on a D4 Advance Diffractometer (λ = 1.5406 Å, Cu Kα radiation, Bruker, Billerica, MA, USA).

### 2.2. Synthesis of NaYF_4_:Yb,Er,Ce Nanoparticles

All the nanoparticles were prepared by a solvothermal process reported elsewhere [5]. In total, 0.20 mmol YbCl_3_, 0.02 mmol ErCl_3_, x% mmol CeCl_3_ (x = 0, 1, 3, 5, 10), (78-x)% mmol YCl_3_, 6 mL OA, and 15 mL ODE were successively added into a 100 mL, three-necked, round-bottomed flask. The whole solution was heated to 130 °C under vacuum until all the powder dissolved. The solution was cooled down to 60 °C and 2.5 mmol NaOH and 1.0 mmol NH_4_F were added. Stirring continued until the NaOH and NH_4_F dissolved. Then, the mixture was heated up to 300 °C and the temperature was maintained for one hour in the N_2_ atmosphere. Finally, the samples were precipitated by excessive ethanol and centrifugation.

### 2.3. Surface Modification of NaYF_4_:Yb,Er,Ce Nanoparticles

The hydrophilic nanoparticles (NaYF_4_:Yb,Er,Ce@SiO_2_-OAlg) were prepared by coating with SiO_2_ and OAlg molecules successively. First, the OAlg molecules were prepared by the oxidation of sodium periodate on sodium alginate [16]. Then, 4 mL ethanol and 0.32 mL TEOS were added into the NaYF_4_:Yb,Er,Ce solution when the pH was adjusted at the 8–9 region. The whole solution was heated in the water bath for 0.5 h at 70 °C. Then, 20 µL APTES solution was dropped and continued to be stirred for 5 h at 70 °C. The NaYF_4_:Yb,Er,Ce@SiO_2_ was obtained by centrifuging for 10 min. Then, OAlg was added and mixed with NaYF_4_:Yb,Er,Ce@SiO_2_ for another 12 h. Finally, the modified nanoparticles were precipitated by centrifugation.

### 2.4. In Vitro Bioimaging of NaYF_4_:Yb,Er,Ce@SiO_2_-OAlg

The HeLa cells were provided by Shanghai Institutes for Biological Sciences, Chi-nese Academy of Sciences. First, the cytotoxicity of NaYF_4_:Yb,Er,Ce@SiO_2_-OAlg was determined through the Cell Counting Kit-8 (CCK-8) process [16]. The Hela cells were put into DMEM supplemented with 10% FBS solution and under the condition of 5% CO_2_ and 37 °C. The cells (10 μg/mL) were placed on a glass slide for 12 h and washed by PBS solution. The cells were incubated with 15 μg/mL NaYF_4_:Yb,Er,Ce@SiO_2_-OAlg for another 3 h. Finally, the UCL images of living Hela cells were obtained on laser scanning upconversion luminescence microscopy (LSUCLM).

### 2.5. Analysis of Absorption and Scattering of NaYF_4_:Yb,Er,Ce@SiO_2_-OAlg

The in vitro experiments were carried out by a standard pattern card. An external 980 nm laser was used as the excitation light. The bottom was filled with the solution of probes, and covered with the card. The optical signals were collected with no tissue cover and covered with 2 mm pork tissue, respectively. A Si-based camera (Andor) and 660 ± 10 nm bandpass filter collected the UCL luminescent images. An NIR camera (Princeton) and 1535 ± 45 nm bandpass filter collected the NIR-II luminescent images. The detection range was “0”, “1”, “2”, and so on groups in the resolution card. Then, in vivo images were obtained in the NIR-II window. The animal experiment was performed in accordance with the norm of the Institutional Animal Care and Use Committee and Animal Ethics Committee of Zhejiang Sci-Tech University. 

## 3. Results

**Figure 2:**
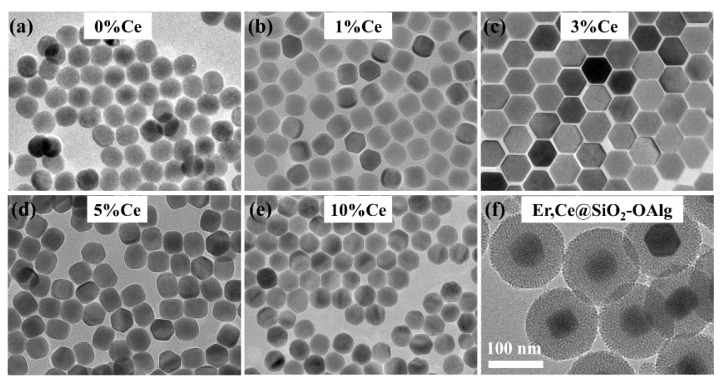
(**a**–**e**) TEM images of different NaYF_4_:20%Yb,2%Er,x%Ce (x = 0, 1, 3, 5, 10) nanoparticles. (**f**) TEM image of the NaYF_4_:20%Yb,2%Er,3%Ce@SiO_2_-OAlg (Er,Ce@SiO_2_-OAlg) nanoparticles. The scale bar is 100 nm.

The TEM images of NaYF_4_:20%Yb,2%Er,x%Ce (x = 0, 1, 3, 5, 10) nanoparticles are shown in Figure 2. The results showed that Ce^3+^-ion-doped nanoparticles have relatively good monodispersity. The concentration of Ce ranges from 0% to 10%, and the size of the obtained nanoparticles ranges from 45–55 nm. With the increase in Ce concentration, the particle size increases slightly, but has little change on shape, with an average diameter of 45 nm. After being coated with silicon shell and modified by OAlg molecules, the nanoparticles are spherical and the diameter is about doubled, which reaches almost 140 nm. The thickness of the SiO_2_ layer is about 45 nm. The elements in the nuclear structure, most of which are rare earth elements, have a large relative atomic mass. Therefore, the area of the core shows a dark color, while the area of the shell shows a gray color because of the small relative atomic mass.

**Figure 3:**
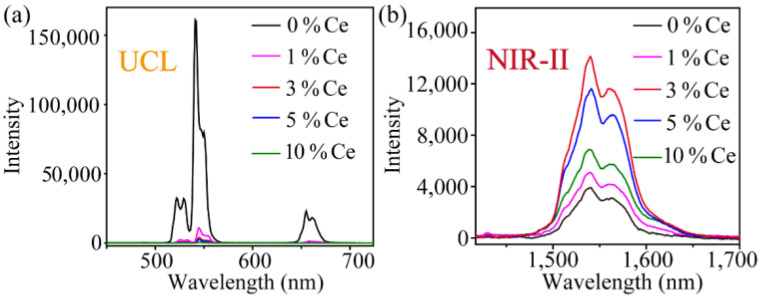
(**a**) Upconversion luminescence spectrum and (**b**) NIR-II luminescence spectrum of NaYF_4_:20%Yb,2%Er,x%Ce (x = 0, 1, 3, 5, 10) nanoparticles (dispersed in cyclohexane).

Subsequently, the upconversion and NIR-II luminescence spectrum of nanoparticles under 980 nm excitation were measured (Figure 3). There are two upconversion emission peaks at 540 nm (^4^S_3/2_ → ^4^I_15/2_) and 660 nm (^4^F_9/2_ → ^4^I_15/2_), respectively (Figure S1). The emission peak intensity at 540 nm is about eight times that at 660 nm. After doping with Ce^3+^, it is accompanied by a sharp decrease in upconversion luminescence with the increased amount of Ce^3+^ ions. In comparison, the intensity of near-infrared luminescence at 1530 nm has enhanced 3.6 times with the increase in Ce^3+^ doping. When the concentration of Ce^3+^ is 3%, the maximum NIR-II luminescence intensity is obtained. This provides a significant reference for the regulation of fluorescence properties of such lanthanide-doped nanoprobes.

The NaYF_4_:Yb,Er,Ce@SiO_2_-OAlg and OAlg have similar characteristic peaks, in which the broadband peak centered at 3421 cm^−^^1^ is due to the stretching vibration of hydrogen bond O-H, and the strong peak at 1612 cm^−^^1^ represents the asymmetric stretching of carboxylic group (-COO-) vibration (Figure S2). The broad absorption band at 1103 cm^−^^1^ is due to the antisymmetric stretching irritation absorption of Si-O-Si. The asymmetric and symmetric stretching vibrational absorption peaks of methylene (-CH_2_-) in the long alkyl chain were found at 2925 cm^−^^1^and 2851 cm^−^^1^. 

The structure of NaYF_4_:Yb,Er,Ce@SiO_2_-OAlg was also determined by XRD (Figure S3). Compared with the standard hexagonal crystal card (JCPDS: 72-2404), the nanoparticles with and without silicon coating are in good agreement. The elements in the nuclear structure, most of which are rare earth elements, have a large relative atomic mass, while shell area shows a gray color. The NaYF_4_:Yb,Er,Ce@SiO_2_-OAlg has a distinct undulating bulge in the range of 20° to 30° that can be attributed to the surface silica coating.

**Figure 4:**
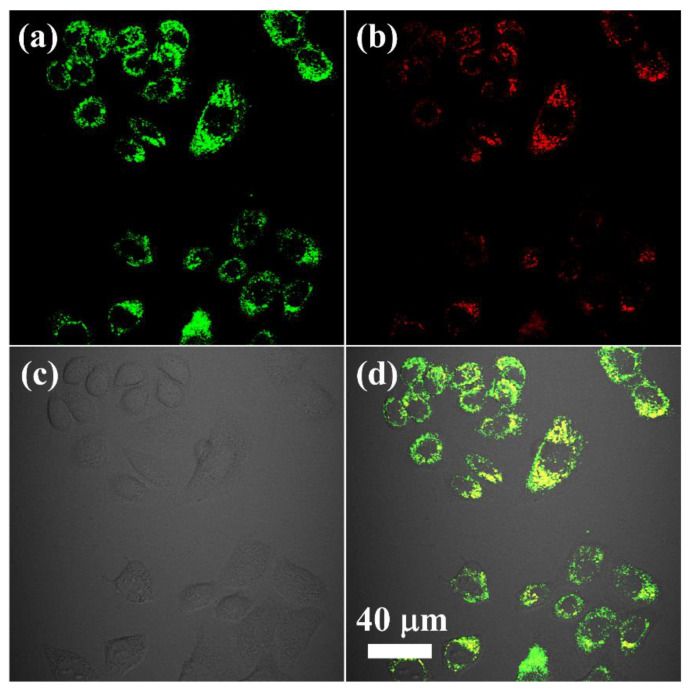
UCL images of living Hela cells after incubation with NaYF_4_:Yb,Er,Ce@SiO_2_-OAlg for three hours. The luminescent images were obtained at a green UCL channel at 500–560 nm (**a**) and a red channel at 600–700 nm (**b**) by a 980 nm laser. (**c**) The image of the bright field of cells. (**d**) Overlay of green UCL and red UCL images. The scale bar is 40 μm.

The cell viability (Figure S4) of nanoparticles was obtained after the incubation with different concentrations of NaYF_4_:Yb,Er,Ce@SiO_2_-OAlg for 24 h, separately. The survival rate of cells was over 80% in high-concentration (800 μg/mL) solution. After incubation with NaYF_4_:Yb,Er,Ce@SiO_2_-OAlg (100 μg/mL) probes for 3 h, the upconversion fluorescent images in living cells were collected under the excitation of a 980 nm laser. As shown in Figure 4, the UCL signals were weaker in the red channel of 600–700 nm, while the apparent signals of UCL are observed in the green channel of 500–560 nm. The intensity contrast of green light and red light can be directly observed by the difference in the overlay image. It showed that the NaYF_4_:Yb,Er,Ce@SiO_2_-OAlg can achieve precise fluorescent imaging at the cellular level.

Unlike fluorescence imaging at the microscopic level of cells, the difficulty of in vivo imaging is the limited penetration depth and spatial resolution of tissues. Here, the R3L3S1N resolution standard card (Figure S5) is used to visually reflect the smallest resolution distance under different conditions (Figure S6). The mask is used to form a pattern of nanoparticles on the surface; then, the 2 mm pork tissue was added on the top. It was determined by the fluorescence signal changes corresponding to each pixel on the line by drawing a vertical line at the three-line pair. Referring to the Rayleigh criterion, if the line width calculated by data is larger than the actual line width, the minimum distance is determined to be indistinguishable. Compared with the resolution measurement method of the V-shaped capillary tube reported previously [30], this method presents the resolution of materials in different luminescent imaging in relatively uniform standards and an intuitive data processing way. It provides a general method worthy of popularization to evaluate the imaging resolution of fluorescent probes.

**Figure 5:**
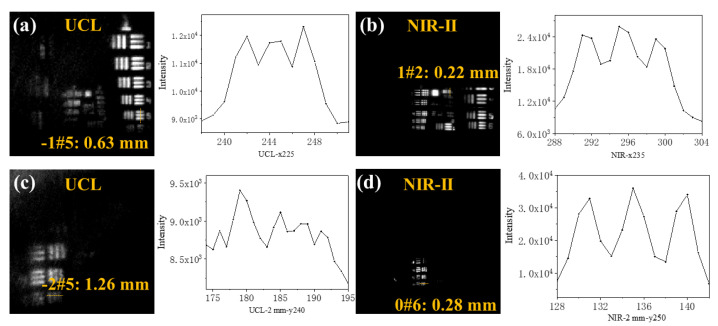
The fluorescence images of nanoparticles were covered with different pork tissues in different fluorescent windows. UCL band (660 ± 10 nm): (**a**,**c**); NIR-II window (1535 ± 45 nm): (**b**,**d**). (**a**,**b**) are covered with no tissue. (**c**,**d**) are covered with 2 mm tissue. Different groups of line pairs correspond to minimum resolutions according to the comparison with the R3L3S1N card. A plot with pixel intensity is shown in the images, along with the corresponding yellow line.

**Figure 6:**
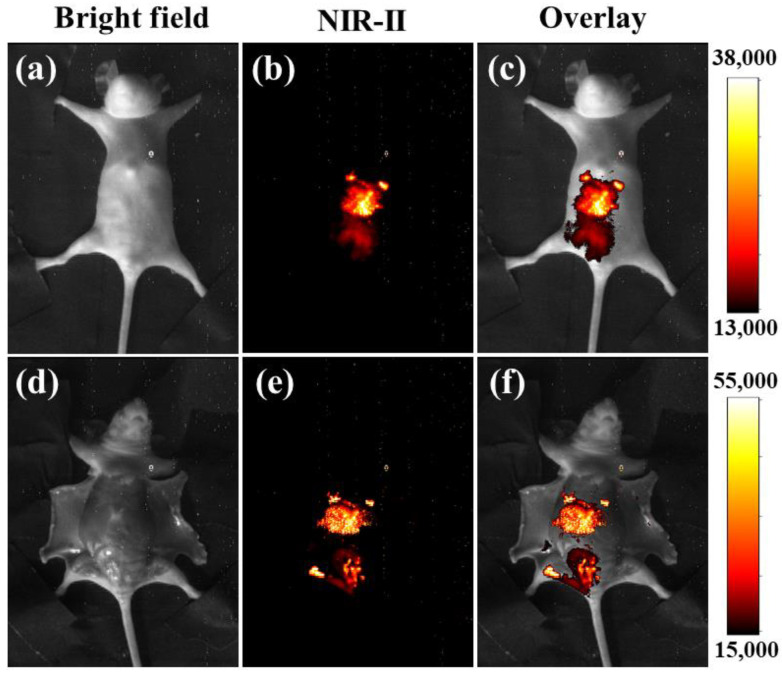
Near-infrared images of mice after injection with NaYF_4_:Yb,Er,Ce@SiO_2_-OAlg. (**a**,**d**) The image of the bright field of mice. (**b**,**e**) The luminescent signals were obtained at the 1490–1580 nm region under excitation by a 980 nm laser. (**c**,**f**) Overlay of the NIR-II and the bright field images.

Compared with the standard resolution distance of the card (R3L3S1N), we took the distance between the two-line pairs that could be recognized as the minimum resolution distance achieved by fluorescence imaging. By collecting the fluorescence imaging data of the upconversion window and the near-infrared window, respectively, the minimum resolution distance can be obtained (Figure 5). When tissues are not covered, the minimum resolution distance of the 660 nm band reaches 0.63 mm, and the minimum resolution distance of the 1530 nm band reaches 0.22 mm. Covering 2 mm biological tissue, the minimum resolution distance of the 660 nm band is 1.26 mm, and the minimum resolution distance of 1530 nm band is 0.28 mm. When the tissue is covered, both of the minimum resolutions are increased; the scattering phenomenon is more severe at 660 nm. The NIR-II imaging is more precise whether there is biological tissue coverage, which is more suitable for animal imaging in vivo. Obviously, the influence of physical background fluorescence is less in the range of NIR-II. Therefore, we chose to collect fluorescent signals at the NIR-II window for in vivo imaging for its much higher resolution (Figure 6). The mice (weight: 19.5 g) were injected with the material (5 mg/mL × 0.1 mL) and placed into the anesthesia chamber of the imaging instrument for the imaging experiment. The concentration has weak toxicity for mice from the cell viability experiment. After injecting into mice via the tail vein for 5 min, the unmistakable NIR-II signals were observed in the liver of mice, indicating good imaging ability of such nanoprobes. Figure 6a–c was obtained before sacrifice and Figure 6d–f was obtained after sacrifice.

## 4. Conclusions

The core-shell structure of NaYF_4_:Yb,Er,Ce@SiO_2_-OAlg was designed and synthesized to improve water solubility and biocompatibility. By gradually increasing the doping amount of Ce^3+^, the luminescence intensity of UCL is suppressed, and the luminescence intensity of NIR-II is grown under the excitation of 980 nm. Subsequently, it can be imaged at the cell level and living level simultaneously, indicating the high resolution, low biological toxicity, bright luminescence, and good imaging effect. This work can be applied to multiscale luminescence imaging in biological research. 

## Supplementary Materials

The following are available online at https://www.mdpi.com/article/10.3390/nano11102676/s1, Figure S1: The energy transform of the NaYF_4_:Yb,Er,Ce-OA and the structure of OAlg molecule. Figure S2: FTIR measurement of Oleic Acid capped NaYF_4_:20%Yb,2%Er,3%Ce nanoparticles (NaYF_4_:Yb,Er,Ce-OA), NaYF_4_:Yb,Er,Ce@SiO_2_ nanoparticles, NaYF_4_:Yb,Er,Ce@SiO_2_-OAlg nanoparticles and the OAlg molecule. Figure S3: XRD measurement of NaYF_4_:Yb,Er,Ce nanoparticles andNaYF_4_:Yb,Er,Ce@SiO_2_-OAlg nanoparticles. Figure S4: The cell toxicity of NaYF_4_:Yb,Er,Ce@SiO_2_-OAlg nanoparticles. Figure S5: The photo of R3L3S1N card from Thorlabs Co., Newton, NJ, USA. Figure S6: 1951 USAF Resolution Target Data from Thorlabs Co., Newton, NJ, USA.
