# Supplementary material for: Er3+-Ions-Doped Multiscale Nanoprobes for Fluorescence Imaging in Cellular and Living Mice
Source: Nanomaterials (Basel). 2021 Oct 12;11(10):2676. doi: 10.3390/nano11102676 (PMC8539509; doi:10.3390/nano11102676)
Supplement: Supplementary file 1 [file nanomaterials-11-02676-s001.zip › nanomaterials-1371623-supplementary.pdf]

Supplementary Materials

Er^{3+} ions doped multiscale nanoprobes for fluorescence imaging in cellular and living mice

Cong Cao*, Yu Xie, Shiwen Li, Chang Hong

Institute of Smart Biomedical Materials, School of Materials Science and Engineering, Zhejiang Sci-Tech University, Hangzhou, 310018, China

*Corresponding author: ccong@zstu.edu.cn (C. Cao)

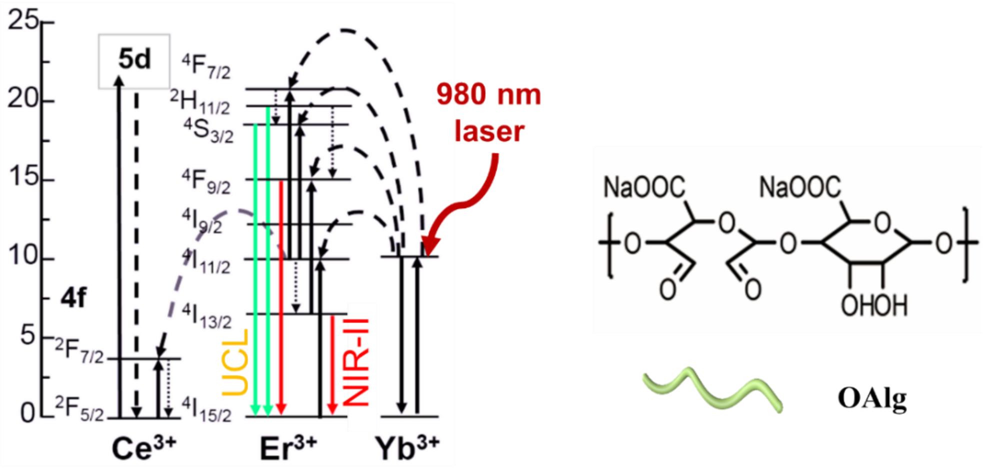

Figure S1. The energy transform of the $NaYF_4$:,Yb,Er,Ce-OA and the structure of OAlg molecule.

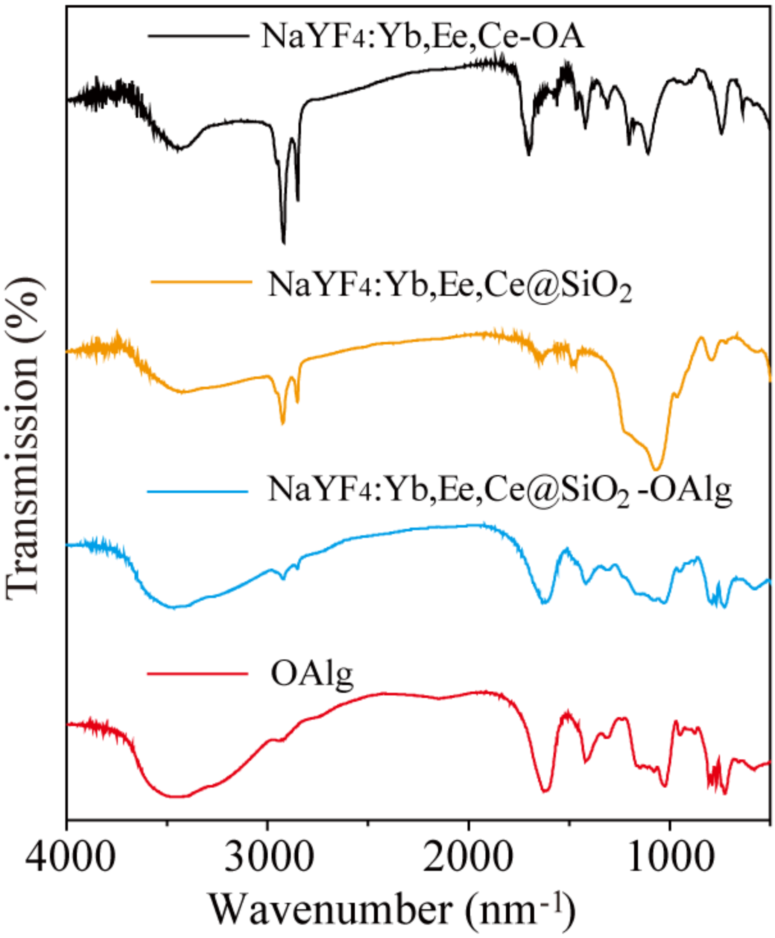

Figure S2. FTIR measurement of $NaYF_4$:20%Yb,2%Er,3%Ce nanoparticles capped with OA ($NaYF_4$:Yb,Er,Ce-OA), $NaYF_4$:,Yb,Er,Ce@SiO_2 nanoparticles, $NaYF_4$:,Yb,Er,Ce@SiO_2-OAlg nanoparticles and the OAlg molecule.

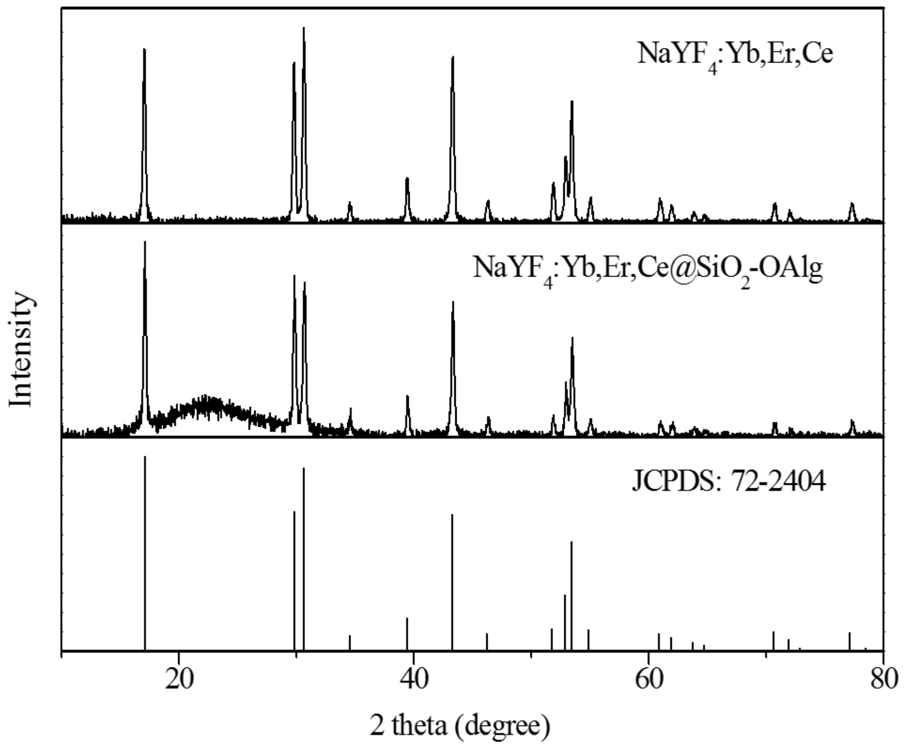

Figure S3. XRD measurement of $NaYF_4$:Yb,Er,Ce nanoparticles and $NaYF_4$:Yb,Er,Ce@SiO_2-OAlg nanoparticles.

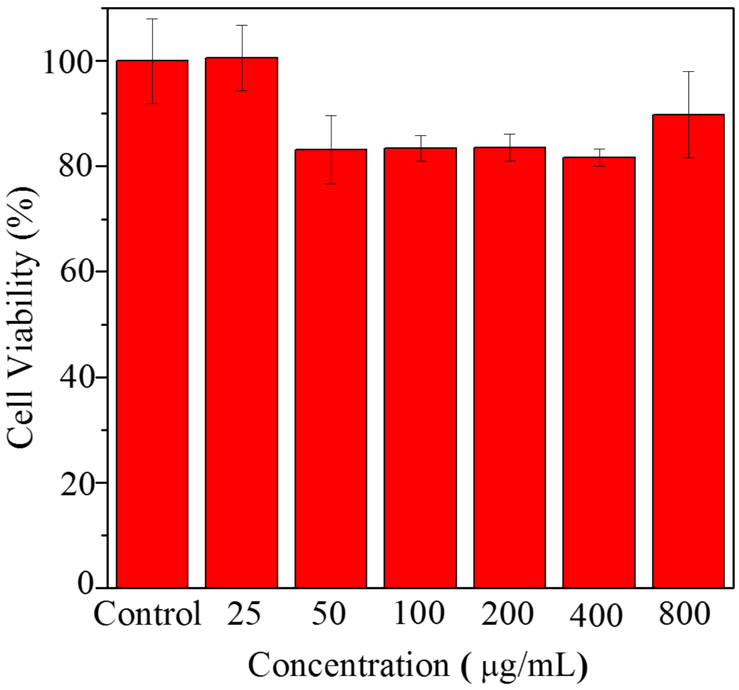

Figure S4. The cell toxicity of $NaYF_4$:Yb,Er,Ce@SiO_2-OAlg nanoparticles.

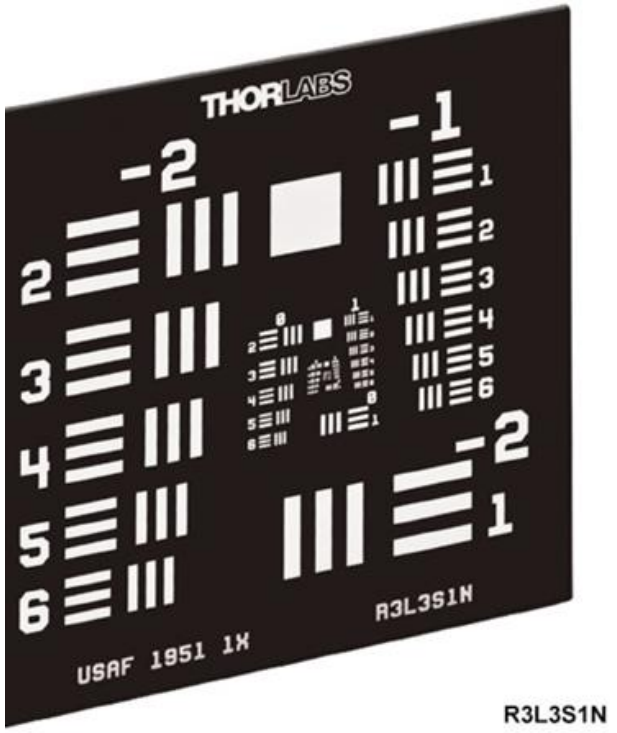

Figure S5. The photo of R3L3S1N card from Thorlabs Co.

Group	Element	Frequency	Line Width (um)
-2	1	0.2500	2000
	2	0.2806	1782
	3	0.3150	1587
	4	0.3536	1414
	5	0.3969	1260
	6	0.4454	1122
-1	1	0.5000	100
	2	0.5612	891
	3	0.6300	794
	4	0.7071	707
	5	0.7937	630
	6	0.8909	561
0	1	1.00	500
	2	1.122	445
	3	1.260	397
	4	1.414	354
	5	1.587	315
	6	1.782	281

Group	Element	Frequency	Line Width (um)
4	1	16.00	31.3
	2	17.96	27.8
	3	20.16	24.8
	4	22.63	22.1
	5	25.40	19.7
	6	28.51	.17.5
5	1	32.00	15.6
	2	35.92	13.9
	3	40.32	12.4
	4	45.25	11.0
	5	50.80	9.84
	6	57.02	8.77
6	1	64.00	7.81
	2	71.84	6.96
	3	80.63	6.20
	4	90.51	5.52
	5	101.6	4.92
	6	114.0	4.38

Group	Element	Frequency	Line Width (um)
1	1	2.000	250
	2	2.245	223
	3	2.520	198
	4	2.828	177
	5	3.174	157
	6	3.564	140
2	1	4.00	125
	2	4.490	111
	3	5.040	99.2
	4	5.657	88.4
	5	6.350	78.7
	6	7.127	70.2
3	1	8.000	62.5
	2	8.980	55.7
	3	10.08	49.6
	4	11.31	44.2
	5	12.70	39.4
	6	14.25	35.1

Group	Element	Frequency	Line Width (um)
7	1	128.0	3.91
	2	143.7	3.48
	3	161.3	3.10
	4	181.0	2.76
	5	203.2	2.46
	6	228.1	2.19
8	1	256.0	1.95
	2	287.4	1.74
	3	322.5	1.55
	4	362.0	1.38
	5	406.4	1.23
	6	456.1	1.10
9	1	512.0	0.977
	2	574.7	0.870
	3	645.1	0.775
	4	724.1	0.691
	5	812.7	0.615
	6	912.3	0.548

Figure S6. 1951 USAF Resolution Target Data from Thorlabs Co.
